# Simultaneous Bilateral Endobronchial Valve Insertion for Management of Persistent Air Leaks Secondary to Necrotising Pneumonia

**DOI:** 10.1002/rcr2.70545

**Published:** 2026-03-11

**Authors:** Jelena Solujic, Michael Brown, Phan Nguyen, Arash Badiei

**Affiliations:** ^1^ Royal Adelaide Hospital Thoracic Medicine Adelaide Australia

**Keywords:** endobronchial valves, necrotising pneumonia, persistent air leak, pneumothorax

## Abstract

Pneumothorax is an abnormal communication between the tracheobronchial tree and the pleural space either as a result of a broncho‐pleural fistula or an alveolo‐pleural fistula. When the air leak from these fistulae persists beyond 5 days it is defined as a persistent air leak (PAL). Whilst surgical repair remains the standard of care, endobronchial valves (EBVs), established for bronchoscopic lung volume reduction (BLVR), are emerging as a minimally invasive alternative. Current data supporting the use of EBVs in PAL are limited to case reports and small retrospective case series. This report details a rare case of bilateral PAL secondary to necrotising pneumonia in a critical ill patient, successfully managed with simultaneous bilateral EBV placement, highlighting the feasibility and clinical efficacy of this approach.

## Graphical abstract

This report details a rare case of bilateral PAL secondary to necrotising pneumonia in a critically ill patient, successfully managed with simultaneous bilateral endobronchial valves placement, highlighting the feasibility and clinical efficacy of this approach.

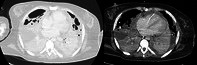

## 1 Introduction

Pneumothorax results from an abnormal communication between the tracheobronchial tree and pleural space, occurring secondary to bronchopleural fistulae or alveopleural fistulae [1]. A pneumothorax with continued air leak greater than 5 days is defined as a persistent air leak (PAL) [1, 2]. PAL is clinically significant, leading to prolonged hospitalisation, increased morbidity and mortality, and associated with complications including pleural infection [1, 2].

Surgical repair remains the gold‐standard for definitive management in patients who are suitable candidates [2]. Endobronchial valves (EBVs) used in bronchoscopic lung volume reduction (BLVR) for patients with severe emphysema, and have emerging evidence for non‐surgical management of PAL. We report a rare case of bilateral PALs secondary to necrotising pneumonia requiring simultaneous bronchoscopic placement of bilateral Pulmonx Zephyr EBVs, to our knowledge there are no other case reports of simultaneous bilateral EBV insertion for PAL.

## 2 Case Report

A 29‐year‐old previously healthy woman presented with acute hypoxic respiratory failure. On admission, she was tachypnoeic and hypoxic with peripheral oxygen saturation of 90% on 4 L/min of supplemental oxygen, improving to 94% on nasal high‐flow oxygen (FiO_2_ 50%, flow 50 L/min). Arterial blood gas on high‐flow oxygen revealed pH 7.43, pO_2_ 60 mmHg, pCO_2_ 30 mmHg and lactate 4.85 mmol/L. Respiratory nucleic acid multiplex panel testing was positive for influenza A. Chest x‐ray demonstrated extensive, bilateral airway opacities in the mid to lower zones. She was admitted to the intensive care unit (ICU) and intubated for progressive hypoxia and acute respiratory distress syndrome (ARDS).

**FIGURE 1:**
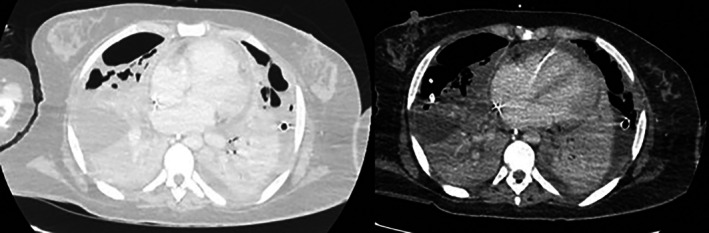
Bilateral pyopneumothoraces (left—lung window, right—soft tissue window).

Her ICU admission was protracted (43 days) and complicated by progressive respiratory failure requiring veno‐venous extracorporeal membrane oxygenation (ECMO). Further complications included septic shock with circulatory failure requiring escalating inotropic and vasopressor support, anuric renal failure necessitating continuous renal replacement therapy, hepatic failure and critical illness polymyoneuropathy. The underlying aetiology included Influenza A infection, ARDS and superimposed methicillin‐resistant staphylococcus aureus (MRSA) necrotising pneumonia, identified radiologically as bilateral consolidation with suspicion of necrosis and abscess formation. Her clinical situation was further complicated by the development of bilateral pyopneumothorax (Figure 1) requiring bilateral chest drain insertion. Pleural fluid sampling was culture positive for *Aspergillus fumigatus* , Acinetobacter and MRSA. Broad‐spectrum antimicrobial treatment included ceftaroline, vancomycin, linezolid, liposomal amphotericin B, voriconazole, amikacin and meropenem.

Despite drainage with a 14 Fr intercostal catheter on the right and 28 Fr intercostal catheter on the left, bilateral air‐leaks persisted. The cardiothoracic surgical and anaesthetic teams deemed her too high‐risk for surgery given her critical condition and bilateral involvement. The respiratory team was therefore consulted for endobronchial management options.

**FIGURE 2:**
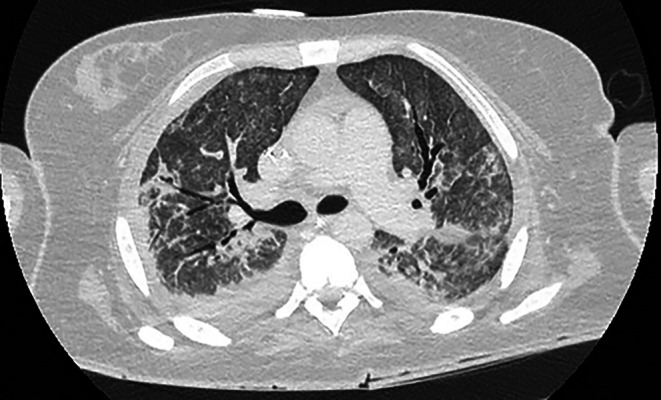
CT chest (lung window) 24 h following insertion of bilateral EBVs showing improvement in aeration.

A bronchoscopy was performed in the ICU. Sequential and systematic balloon isolation was performed from the main stem bronchi to segmental airways using a combination of Chartis (Pulmonx, Redwood, CA) and standard Fogarty balloons, with concurrent observation of the underwater seal drains to localise the leaks. Cessation of bubbling was confined to the isolation and occlusion of the lingula and right middle lobe (RML). Two large (5.5 mm), regular length Zephy EBVs (Pulmonx, Redwood, CA) were inserted into both the lingula and RML, respectively. There was immediate visual cessation of bubbling from the bilateral chest drains. A repeat CT performed within 24 h demonstrated complete resolution of the pneumothoraces and improvement in aeration of the lungs (Figure 2).

There were no procedure‐related complications. Following valve insertion, the patient's condition stabilised sufficiently to allow for surgical video‐assisted thoracoscopy for infection source control. Unfortunately, despite these efforts, the patient died due to overwhelming sepsis and multi‐organ failure.

## 3 Discussion

Necrotising pneumonia complicated by pneumothorax and PAL in critically unwell patients is a challenging clinical problem. These patients typically have limited respiratory reserve and are often poor surgical candidates [3]. The PAL and continuous airflow through the fistula delays healing, contaminates the pleural space, and restricts the use of positive end‐expiratory pressure during ventilation [1]. One‐way EBVs offer a minimally invasive solution to control air leak in carefully selected patients.

EBVs are indicated for BLVR in selected patients with severe emphysema and hyperinflation [4]. There are two commercially available valves: the Zephyr EBV (Pulmonx, Redwood City, CA) and the Spiration intrabronchial valve (IBV) (Olympus Respiratory America, Redmond, WA). These valves provide a reversible option for PAL management, differentiating them from other approaches such as blood patch and chemical sclerosants (via chest drain) [1]. The one‐way valves allow distal secretions to drain normally, unlike Watanabe spigots, while preventing air entry, making them particularly advantageous in the context of pulmonary sepsis [1]. Successful EBV insertion relies on air leak isolation, valve and airway sizing and accurate deployment [2].

Evidence supporting the use of EBVs for the management of PALs remains limited, consisting predominantly of case reports and small retrospective case series [3]. The most recent clinical practice guidelines from the British Thoracic Society highlight the need for further high‐quality evidence to establish the optimal indications and timing of EBV therapy [5].

This case describes an exceptionally complex clinical scenario involving bilateral PALs secondary to necrotising pneumonia, complicated by ARDS and multiorgan failure requiring advanced intensive care support. Despite these challenges, EBV insertion was successfully performed while the patient was supported with ECMO, demonstrating the feasibility of this approach in a high‐risk setting. The intervention resulted in immediate cessation of both air leaks and complete radiological resolution within 24 h, eliminating the need for high‐risk surgery for the pneumothoraces. Given the time critical nature of the patient's rapidly declining clinical trajectory, the goal of reducing or stopping the PAL rather than achieving lobar collapse, and the patient being supported by ECMO, treatment of the PAL was undertaken in consultation with the intensive care team in a single procedure rather than a staggered approach. This case highlights that in appropriately selected patients, systematic balloon isolation and placement of EBVs may have a role in critically ill patients with pneumothorax complicated by PAL.

In conclusion endobronchial valve treatment can provide a minimally invasive treatment option for PALs in critically unwell patients unsuitable for surgery. This case uniquely highlights the feasibility and efficacy of this option in a critically unwell patient. Further study is warranted to define the optimal indications and timing of EBV insertion.

## Author Contributions

**Jelena Solujic:** procedure, patient care and follow up, primary author of article. **Michael Brown:** preparation and review of article. **Phan Nguyen:** preparation and review of article. **Arash Badiei:** procedure supervisor, patient care and follow up, preparation and review of article, case report supervisor.

## Funding

The authors have nothing to report.

## Consent

The authors declare that written informed consent was obtained for the publication of this manuscript and accompanying images and attest that the form used to obtain consent from the patient complies with the Journal requirements as outlined in the author guidelines.

## Conflicts of Interest

Phan Nguyen is an Editorial Board member of Respirology Case Reports and a co‐author of this article. He was excluded from all editorial decision‐making related to the acceptance of this article for publication. The other authors declare no conflicts of interest.

## Data Availability

The data that support the findings of this study are available on request from the corresponding author. The data are not publicly available due to privacy or ethical restrictions.
